# Transcriptional reprogramming and co-expression network underlying enhanced ammonium uptake in intraspecific hybrids of *Saccharum* sp*ontaneum*

**DOI:** 10.3389/fpls.2026.1758719

**Published:** 2026-02-25

**Authors:** Feiyan Zhao, Sisi Zhang, Yinyong Li, Ting Yang, Zongtao Yang, Jiayong Liu, Fenggang Zan, Jun Deng, Jianmin Wu, Yong Zhao, Zuhu Deng, Xinwang Zhao, Yuebin Zhang

**Affiliations:** 1 National Engineering Research Center for Sugarcane, Fujian Agriculture and Forestry University, Fuzhou, Fujian, China; 2 Sugarcane Research Institute, Yunnan Academy of Agricultural Sciences, Kaiyuan, Yunnan, China; 3 Sugarcane Research Institute, Guangxi Academy of Agricultural Sciences, Nanning, China

**Keywords:** nitrogen-efficient, *Saccharum spontaneum*, sugarcane, transcriptome, WGCNA

## Abstract

**Introduction:**

In sugarcane production, nitrogen utilization efficiency is generally suboptimal, averaging only 30–40%. *Saccharum spontaneum*, the wild progenitor of sugarcane, harbors abundant genetic resources for high nitrogen efficiency, which remain largely untapped. Notably, the application of intraspecific hybridization in *S. spontaneum* for improving nitrogen efficiency in sugarcane breeding remains unexplored.

**Methods:**

Against this background, in March 2024, an initial investigation was conducted at the Sugarcane Research Institute of the Yunnan Academy of Agricultural Sciences, China, focusing on four *S. spontaneum* germplasm materials (YN82, GSM22, GSM12, YN2) and two intraspecific F1 hybrids (A2, B1), to explore the mechanisms underlying heterosis in nitrogen use efficiency in *S. spontaneum*.

**Results:**

Physiological assays revealed that the hybrids specifically enhanced ammonium (^15^NH_4_^+^) uptake capacity but not nitrate uptake. Comparative transcriptomics and weighted gene co-expression network analysis (WGCNA) unveiled a systemic transcriptional reprogramming in hybrids. This reprogramming involved the coordinated downregulation of nitrate assimilation genes and the rewiring of starch/sucrose metabolism, facilitating carbon skeleton supply for energetically favorable ammonium assimilation. WGCNA identified key modules significantly correlated with ammonium uptake. From these modules, we pinpointed 14 core candidate genes constituting a multi-layered regulatory network, encompassing transcription factors (e.g., AP2/EREBP, bHLH, MYB), nitrogen assimilation enzymes (*GAD*), carbon metabolism providers (*TPP*, *TPS*), and root development regulators (*HCT*, *CYP84A1*).

**Discussion:**

Our work deciphers how intraspecific hybridization triggers systemic optimization to improve NUE and provides novel gene resources for breeding nitrogen-efficient sugarcane.

## 1 Introduction

Sugarcane is an important crop for sugar production and energy purposes, yet its nitrogen use efficiency (NUE) has remained relatively low, ranging from approximately 30% to 40% (Brackin et al., 2015; Ma et al., 2024). “Nitrogen use efficiency (NUE)” typically refers to biomass/yield per unit N. Among these, nitrogen uptake efficiency constitutes the primary determinant of overall nitrogen use efficiency—the limited capacity of crops to absorb applied soil nitrogen represents a critical factor contributing to nitrogen fertilizer loss and suboptimal utilization rates. Excessive nitrogen application not only increases production costs but also leads to a series of severe environmental issues, such as water eutrophication, soil compaction, and greenhouse gas emissions (Vieira-Megda et al., 2015; Otto et al., 2016; Thorburn et al., 2017; Wang et al., 2023). Therefore, tapping into and leveraging the genetic potential of crops for efficient nitrogen uptake and utilization, particularly enhancing nitrogen uptake efficiency, represents a crucial strategy for achieving sustainable agricultural development (Anas et al., 2020; Li et al., 2025).

As an important wild progenitor of modern sugarcane cultivars, *S.* sp*ontaneum* possesses a robust root system, exceptional resistance to both biotic and abiotic stresses, and extensive genetic diversity. It is widely regarded as an indispensable genetic reservoir for sugarcane improvement (Anas et al., 2021; Kiet et al., 2025; Wang et al., 2025) have shown that the *S.* sp*ontaneum* subgenome contributes key genes to the high nitrogen efficiency phenotype of modern sugarcane. These genes are extensively involved in various biological pathways, including nitrogen metabolism, carbohydrate metabolism, photosynthesis, and amino acid metabolism (Anas et al., 2021; Luo et al., 2023; Kiet et al., 2025). In traditional breeding, *S.* sp*ontaneum* is commonly utilized as a male parent in distant hybridization to introduce resistance genes into cultivated varieties (Srivastava et al., 1994; Deng, 2012). However, the extensive genetic variation within this species, particularly the superior alleles associated with nitrogen efficiency, has yet to be fully explored and integrated. Notably, intraspecific hybridization in rice has been shown to enhance resistance against pests such as the Sogatella furcifera, Nilaparvata lugens, and Scirpophaga incertulas, while concurrently conferring heterosis in yield and yield-related traits (Sreewongchai et al., 2021; Horgan et al., 2024). The phenomenon of heterosis in intraspecific hybridization of Arabidopsis thaliana is also widely documented (Groszmann et al., 2014; Le et al., 2023). For instance, synchronizing flowering time through overexpression of the florigen gene (FT) can more clearly elucidate the intrinsic heterosis resulting from heterozygosity (Yamaguchi et al., 2023). This phenomenon is observed across multiple crops, such as maize (Altaweel and Asst.Prof, 2021; Schultz et al., 2023), cotton (Li et al., 2022, Li et al., 2023; Zhang et al., 2023), rapeseed (Ma et al., 2023), and melon (Dafna et al., 2021). Such intraspecific hybridization, which harnesses heterosis by integrating favorable alleles, has been demonstrated as an effective strategy for improving complex traits through efficient genetic improvement. Given the abundance of genetic variations associated with stress resistance and high metabolic efficiency inherent in *S.* sp*ontaneum*, we hypothesize that intraspecific hybridization represents a viable approach to enhance nitrogen use efficiency. However, research on germplasm resources of *S.* sp*ontaneum* and intraspecific hybridization for optimizing its nitrogen uptake strategies remains markedly insufficient (Liang et al., 2022; Healey et al., 2024; X et al., 2025). Consequently, this study aims to investigate the potential contributions of intraspecific hybridization in *S.* sp*ontaneum* and its associated composites to high-nitrogen breeding in sugarcane. By screening and developing germplasm resources with high nitrogen uptake efficiency, this research provides novel insights and material foundations for sustainable sugarcane breeding. sp

Carbon (C) and nitrogen (N) are indispensable core elements for plant life activities, and the coordination of their metabolic and transport networks is crucial in determining crop growth and yield (Coruzzi and Zhou, 2001). Carbon metabolism not only provides the essential carbon skeletons for nitrogen assimilation but also supplies energy (e.g., ATP and NADPH) (Chang et al., 2025). Meanwhile, nitrogen is a critical component of photosynthetic structures such as chloroplasts and Rubisco (Nunes-Nesi et al., 2010). This tight coupling indicates that any strategy aimed at improving crop nitrogen use efficiency (NUE) must account for its synergy with carbon metabolism rather than treating them as isolated systems (Lu et al., 2019; Liu et al., 2025), deciphering the coordinated regulatory networks of carbon and nitrogen metabolism has become a forefront focus in crop genetic improvement (Yang et al., 2016; Amoah et al., 2025; Zou et al., 2025). Interestingly, this synergistic interaction may naturally exist and operate more efficiently in certain wild germplasm resources (Wang et al., 2024). Preliminary observations have revealed that hybrid progeny within *S.* sp*ontaneum* exhibit heterosis in traits such as biomass. This raises a key scientific question: Could the aggregation of *S.* sp*ontaneum* lineage trigger systematic transcriptional reprogramming, thereby synergistically optimizing multiple pathways, including carbon and nitrogen metabolism, to simultaneously enhance nitrogen absorption efficiency effectively?

We note that this study is designed as a mechanistic exploration of intra-specific hybridization potential. Accordingly, the four *S.* sp*ontaneum* accessions were selected as parents based on their contrasting agronomic traits (e.g., maturation period) to provide a clear phenotypic framework for testing heterosis, rather than to encompass the full genetic diversity of the species. Thus, this work serves primarily as a proof-of-concept, providing foundational insights into this underexplored strategy. Furthermore, to enable precise phenotyping and mechanistic dissection, the experiment was conducted under controlled greenhouse/pot conditions at the seedling stage. While this approach was critical for isolating the effects of hybridization on nitrogen uptake physiology and gene expression, the translation of these findings to field performance and yield necessitates future validation through multi-location field trials within a breeding pipeline.

We hypothesize that intraspecific hybridization can systemically reprogram carbon and nitrogen metabolic networks, leading to a more efficient nitrogen acquisition strategy. To investigate this hypothesis, we developed intraspecific hybrid F_1_ progeny from a cross between four *S.* sp*ontaneum* accessions. These parents were chosen as contrasting phenotypes exhibiting significant variation (*P* < 0.05, one-way ANOVA) across multiple key agronomic traits, including plant height, stalk diameter, and single-stalk weight. This deliberate selection aimed to maximize the phenotypic and genetic contrast for this mechanistic study, rather than to represent the full diversity of the species. Therefore, this study intends to investigate the formation mechanism of heterosis resulting from intraspecific hybridization in *S.* sp*ontaneum* concerning nitrogen uptake at the transcriptomic level. This study aimed to: (1) Physiologically characterize the specificity of nitrogen source uptake in hybrids; (2) Decipher the genome-wide transcriptional reprogramming induced by hybridization; (3) Investigate the coordinated changes in carbon and nitrogen metabolism pathways; and (4) Through weighted gene co-expression network analysis (WGCNA), core regulatory genes and networks associated with the observed heterosis in ammonium uptake were identified. Our findings provide novel insights into the mechanisms underlying heterosis in nitrogen use efficiency. The key genes screened can be directionally optimized via gene editing techniques to enhance nitrogen efficiency in sugarcane cultivars, thereby establishing a valuable genetic resource repository for molecular breeding.

## 2 Materials and methods

### 2.1 Plant materials

This research was initiated in March 2024 at the Sugarcane Research Institute of the Yunnan Academy of Agricultural Sciences, located in the Honghe Hani and Yi Autonomous Prefecture, Yunnan Province, China. Based on phenotypic variation in key agronomic traits (as discussed in the Introduction section), four *S.* sp*ontaneum* accessions were selected as parents to maximize genetic divergence, thereby establishing a foundation for investigating the mechanisms underlying heterosis. Six sugarcane materials used in this study were kindly provided by the Sugarcane Research Institute of Yunnan Academy of Agricultural Sciences (**Table 1**). These included four accessions of *S.* sp*ontaneum* (YN82, GSM22, GSM12, YN2) and two hybrid cultivars (A2, B1).

**Table 1:** Genetic backgrounds of 6 sugarcane materials.

Material numbers	Material code	Material type	Parental cross (female × male)
1	YN2	*Saccharum* sp*ontaneum*	–
2	YN82	*Saccharum* sp*ontaneum*	–
3	GSM12	*Saccharum* sp*ontaneum*	–
4	GSM22	*Saccharum* sp*ontaneum*	–
5	A2	hybrid	YN82×GSM22
6	B1	hybrid	YN2×GSM12

### 2.2 Planting materials

All materials were cultivated using the pot-growing method. Fresh soil samples were used as the substrate, which were thoroughly mixed and sieved through a 2 mm mesh to remove large soil fauna and plant residues. Each pot was filled with 10 kg of soil and planted with six buds. Planting was carried out in mid-March 2024. The experiment comprised six *S.* sp*ontaneum* accessions. Each accession was arranged with five pots constituting one experimental unit. A completely randomized block design was adopted with three replicates, resulting in 15 pots per accession and 90 pots in total for all six accessions. Pots were spaced 0.8 m apart, with each row and column bordered by the cultivated sugarcane variety ‘YZ05-51’ as a control. Field management followed standard agronomic practices.

The pot served as the basic experimental unit. To ensure statistical independence, a composite sampling approach was employed: during the rapid growth stage, one root sample was randomly collected from each pot within a replicate (i.e., an experimental unit of five pots). The five subsamples were combined into one composite sample for analysis. Each accession thus yielded three independent biological replicates (n=3), each corresponding to a distinct experimental unit. This design effectively avoids pseudoreplication within pots and allows robust evaluation of differences among accessions.

### 2.3 ^15^N isotope absorption experiments

At the sugarcane seedling stage, around the 5-leaf stage to the early tillering stage, six uniformly growing plants were selected for each material (two plants per plot) and randomly divided into two treatment groups. Each group consisted of three plants as biological replicates. Each plant was irrigated with 500 mL of ¹^5^N-labeled (¹^5^NH_4_)_2_SO_4_ or K¹^5^NO_3_ solution at a concentration of 11 mmol/L. After 72 hours of treatment, root samples were collected, thoroughly rinsed with deionized water, and subjected to enzyme inactivation at 105 °C for 30 minutes. The samples were then dried to a constant weight in an oven at 75 °C. The dried samples were ground and analyzed for ¹^5^N atom% excess using an elemental analyzer-stable isotope ratio mass spectrometer (EA-IRMS).

### 2.4 Transcriptome sampling, sequencing, and data processing

Root maturation zones (1–3 cm from the root tip) were collected for all six materials (YN82, GSM22, GSM12, YN2, A2, B1) during the rapid growth stage. For each material, samples from five buckets were pooled into a single replicate, with three replicates set up, resulting in a total of 18 samples. These samples were rapidly frozen in liquid nitrogen and stored at -80 °C.

Total RNA was extracted using TRIzol^®^ Reagent, with quality and concentration assessed using the 5300 Bioanalyzer (Agilent) and ND-2000 (NanoDrop Technologies). All RNA samples met the library construction criteria (concentration ≥ 20 ng/μL, total quantity >1 μg). Sequencing libraries were prepared by Shanghai Majorbio Biopharm Technology Co., Ltd. using the Illumina^®^ Stranded mRNA Prep, Ligation kit and sequenced on the NovaSeq X Plus platform with 150 bp paired-end (PE150) reads. On average, approximately 44 million high-quality clean reads were obtained per sample (complete data provided in **Supplementary Table S1**).

Raw sequencing data underwent quality control and filtering using Fastp (v0.23.2). Clean reads were aligned to the sugarcane reference genome AP85-441(https://sugarcane.gxu.edu.cn/scdb/genomics/genome/ap85) using HISAT2 (v2.2.1) under default parameters. Subsequently, reference-guided transcript assembly was performed using StringTie (v2.2.1), and raw read counts for each gene were generated for downstream analyzes.

Differential expression analysis was conducted based on the raw count data using the R package DESeq2 (v3.5.2). Genes with |log2 (fold change)| ≥ 1 and a false discovery rate (FDR) < 0.05 were considered differentially expressed.

Gene Ontology (GO) functional enrichment and Kyoto Encyclopedia of Genes and Genomes (KEGG) pathway enrichment analyzes of the identified differentially expressed genes were performed using the R package clusterProfiler (v4.2.2). A threshold of adjusted P-value (Padjust) < 0.05 was used to define significant enrichment, with the top 20 significantly enriched pathways presented.

### 2.5 Weighted gene co-expression network analysis

To construct a gene co-expression network, the R package Weighted Gene Co-expression Network Analysis (WGCNA) was employed. Genes exhibiting low expression levels (mean FPKM < 1) or low variability (coefficient of variation < 0.1) across all samples were excluded prior to analysis. A scale-free topology was achieved using a soft-thresholding power of β = 12, and co-expression modules were detected using the dynamic tree cut algorithm. Module eigengenes (MEs) were correlated with phenotypic traits, and modules showing |r| > 0.7 and P < 0.05 were identified as significantly associated with the traits of interest. Within these key modules, the top 30 most highly connected genes were selected as candidate hub genes, and their interaction networks were visualized using OmicShare.

### 2.6 Weighted RT-qPCR validation

To validate the accuracy of the transcriptome sequencing results, eight genes were randomly selected from the significantly differentially expressed genes for RT-qPCR validation. Primers were designed using Primer-NCBI (**Table 2**), with GAPDH serving as the internal reference gene (Ling et al., 2014). Amplification was performed using the SYBR^®^ Premix Ex Taq™ II kit on the ABI7500 FAST system. The reaction conditions were as follows: 95 °C for 30 seconds; 95 °C for 5 seconds, 60 °C for 34 seconds, for a total of 45 cycles. Each sample was tested in triplicate, and relative expression levels were calculated using the 2^-ΔΔCt^ method (Livak and Schmittgen, 2001).

**Table 2:** Primers used for RT-qPCR.

Gene ID/Internal reference name	Forward primer (5’-3’)	Reverse primer (5’-3’)
Sspon.01G0005910-1A	TCTGACCTGACTGACGACCC	CTGCACCAGGCCTATGAACT
Sspon.01G0008670-1A	TGGATCAGAGCGTGGTGTTC	GACCCAAAGCCCAGGTTCTT
Sspon.01G0014520-1A	ACCTTGTTTCTGGCGTTGGA	GTTGGTGAAGAGCTCGGACA
Sspon.01G0036400-3D	CGCCCCTGACGATTGTATGT	ACCTTCGGAGAGCAAAGAGC
Sspon.01G0042790-2C	AGATGCTCGGTCAAAAGGCA	TGGTACAAACCTGCCCAGTC
Sspon.02G0000920-1T	AGCTTTTCAGCAAGTCCCCA	GCGAGACACGTAGCCTTCTT
Sspon.02G0000920-4D	GGAACCGTGTCTCTGAGCAA	CTGTACCACCGGCATCACTT
Sspon.06G0023550-1B	ATCCACGTCAGATGCGAGTC	ACCTGCTATCGGCATGTGAG
*GAPDH*	CACGGCCACTGGAAGCA	TCCTCAGGGTTCCTGATGCC

### 2.7 Data analysis

The statistical analysis of the ^15^N isotope absorption experiment data was performed using R software. The significance of differences in nitrogen absorption characteristics among different materials was tested using one-way analysis of variance (One-way ANOVA), with a threshold of P < 0.05 considered statistically significant.

## 3 Results

### 3.1 Analysis of nitrogen uptake characteristics in *Saccharum* sp*ontaneum* parents and their F_1_ progeny

**Figure 1:**
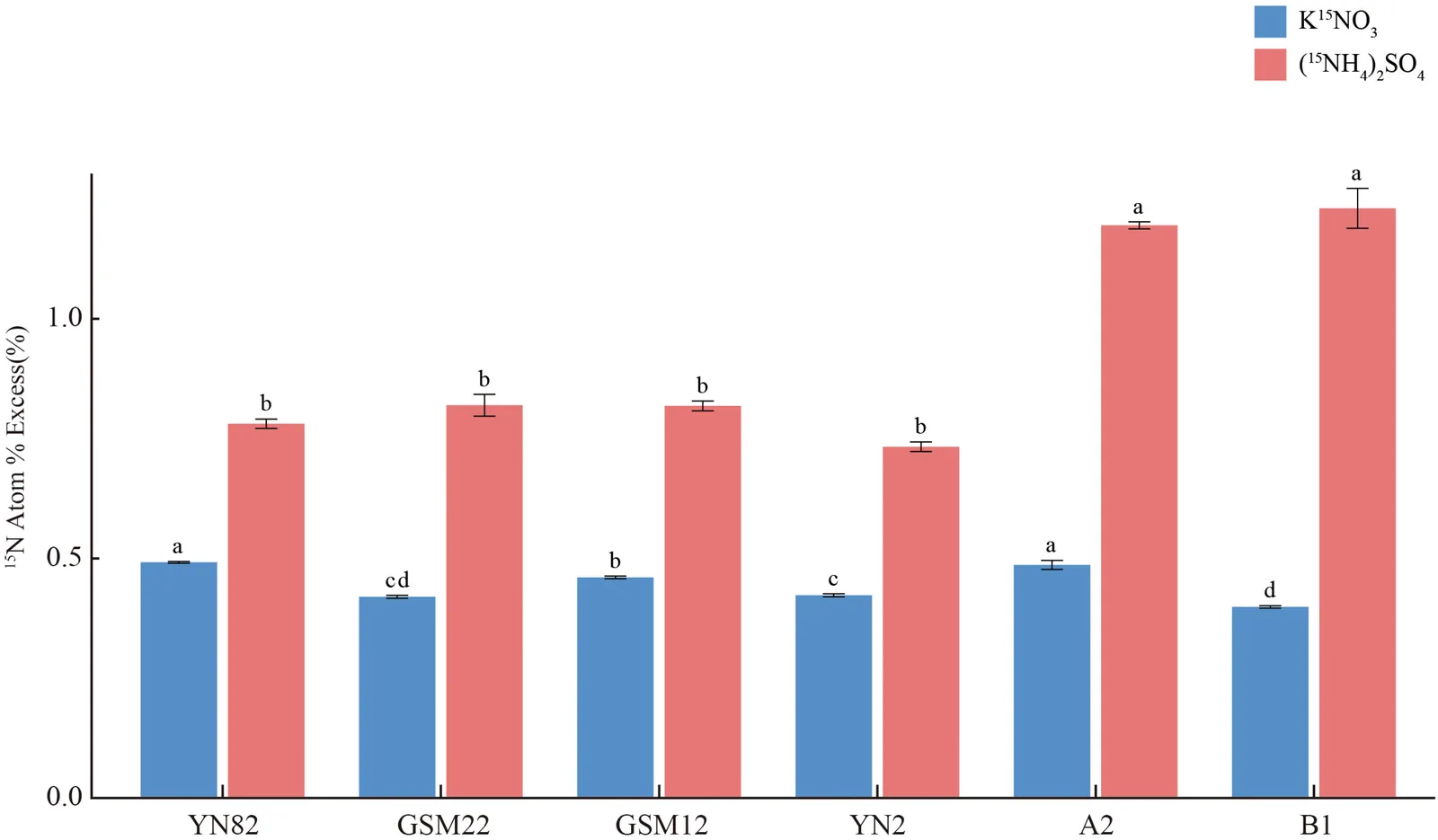
Nitrogen uptake kinetics in roots of the six sugarcane materials. Data are presented as mean ± SD (n = 3). Statistical significance was determined by one-way ANOVA, and different letters above the bars indicate significant differences at *P* < 0.05.

To investigate the effect of intraspecific hybridization in *S.* sp*ontaneum* on nitrogen uptake capacity, we evaluated the root nitrogen uptake characteristics of four *S.* sp*ontaneum* parents (YN82, GSM22, GSM12, YN2) and their two F_1_ progeny, A2 (YN82 × GSM22) and B1 (YN2 × GSM12). As shown in **Figure 1**, the F_1_ progeny A2 and B1 did not exhibit a significant increase in root atom percent excess of ¹^5^N-labeled nitrate compared to the parents. In contrast, the atom percent excess of ¹^5^N-labeled ammonium was significantly higher in both F_1_ hybrids than in the parental lines (*P* < 0.05). These results indicate that intraspecific hybridization specifically enhances the ammonium nitrogen uptake capacity in the offspring.

### 3.2 Transcriptome sequencing

To elucidate the molecular mechanism of high-efficiency nitrogen absorption in the F_1_ generation of *S.* sp*ontaneum*, we prepared 18 cDNA libraries for RNA-seq analysis. After conducting rigorous quality control on the sequencing data, we obtained a total of 45.92 GB of data. The Q30 ratio exceeded 95.33% (95.33%-96.40%), and the GC content ranged from 52.50% to 55.93% (**Supplementary Table 1**).

### 3.3 Analysis and functional annotation of DEGs

**Figure 2:**
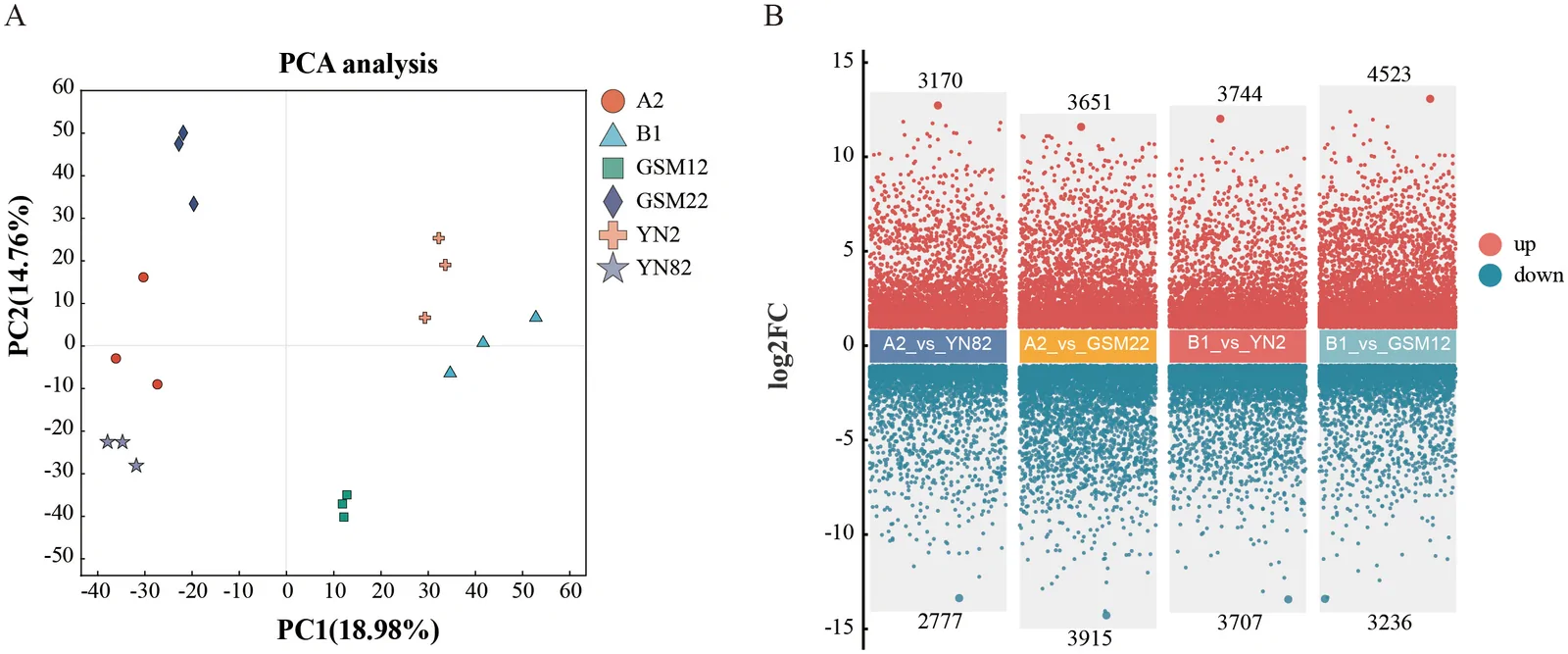
Transcriptome sequencing analysis. **(A)** Principal Component Analysis (PCA): Demonstrating the separation of samples based on transcriptome profiles. **(B)** Volcano Plot: Illustrating differential gene expression between comparison groups. Each point on the volcano map represents a gene, with green and red points representing down-regulated and up-regulated genes, respectively.

The principal component analysis (PCA) results reveal a clear separation of different genotypes at the transcriptome level (**Figure 2A**). The first principal component (PC1) and the second principal component (PC2) explain 18.98% and 14.76% of the total variance, respectively, indicating significant differences in gene expression among the genotypes. Further differential expression analysis (**Figure 2B**) identified 5,947 differentially expressed genes (DEGs) in the A2 vs. YN82 comparison, including 3,170 up-regulated genes and 2,777 down-regulated genes. In the A2 vs. GSM22 comparison, the number of DEGs increased to 7,566, with 3,651 up-regulated and 3,915 down-regulated genes. Similarly, 7,451 DEGs (3,744 up-regulated and 3,707 down-regulated) and 7,759 DEGs (4,523 up-regulated and 3,236 down-regulated) were detected in the B1 vs. YN2 and B1 vs. GSM12 comparisons, respectively. These consistent results demonstrate that the hybridization process triggered extensive remodeling of gene expression regulation in the F1 generation.

### 3.4 Functional enrichment analysis of differentially expressed genes

**Figure 3:**
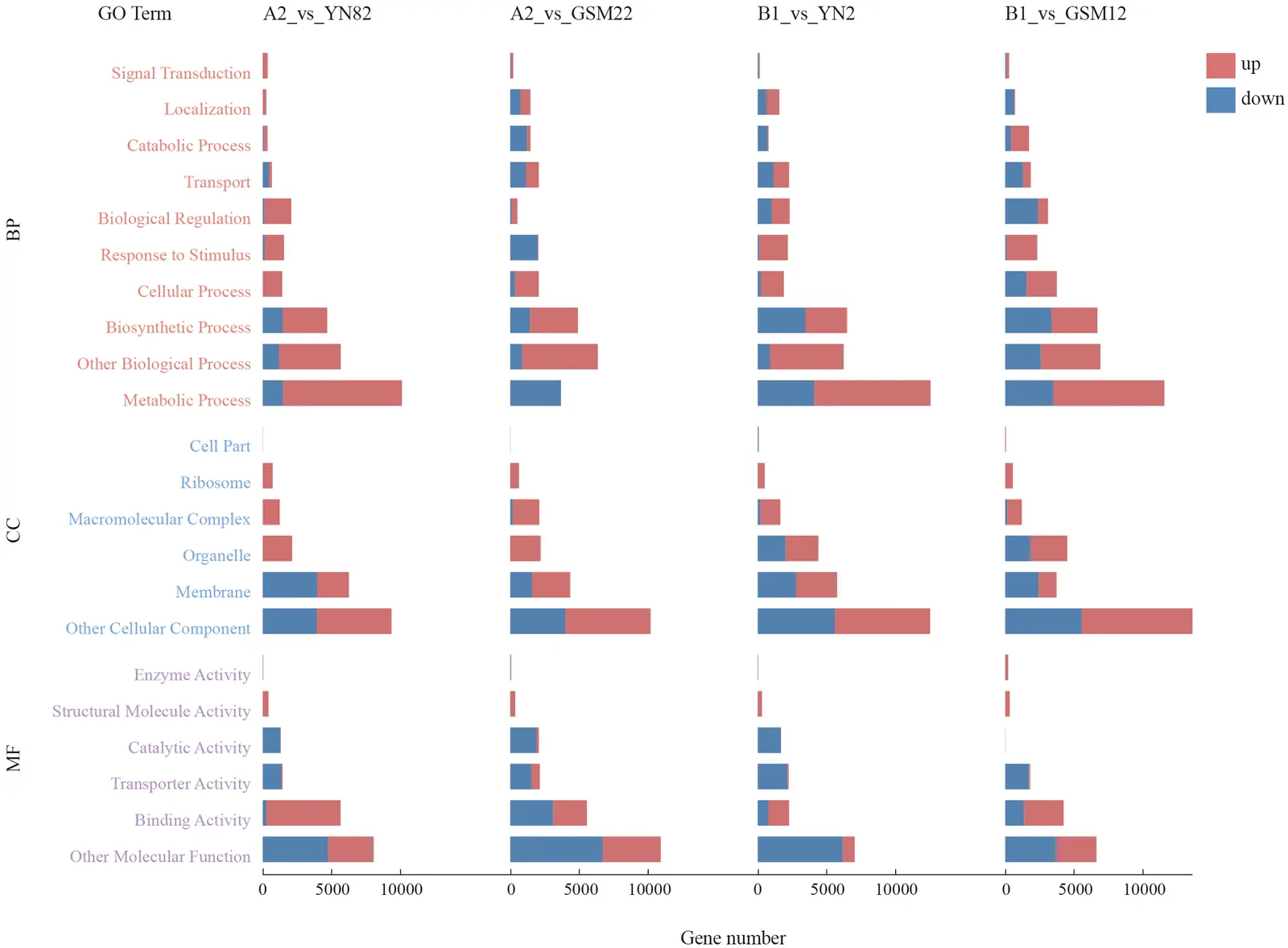
GO enrichment analysis of differentially expressed genes (DEGs) between the hybrid F_1_ generation and parental lines. Red indicates upregulated DEGs in the F_1_ generation compared to the parental lines, while blue represents downregulated DEGs.

To further analyze the functional roles of differentially expressed genes (DEGs) in the four comparison groups (A2 vs. YN82, A2 vs. GSM22, B1 vs. YN2, B1 vs. GSM12), this study conducted GO and KEGG enrichment analyzes for each group. The GO enrichment analysis revealed that the DEGs in the four comparison groups were enriched in the categories of biological process (BP), cellular component (CC), and molecular function (MF) (**Figure 3**). Among these, terms such as water transmembrane transporter activity (GO:0005372), aquaporin activity (GO:0015250), and protein heterotetramerization (GO:0051290) had the highest number of enriched genes. This suggests that the hybridization process may have broadly influenced the material transport capacity of root cells and the formation of protein complexes, laying a foundation for systematic physiological optimization.

**Figure 4:**
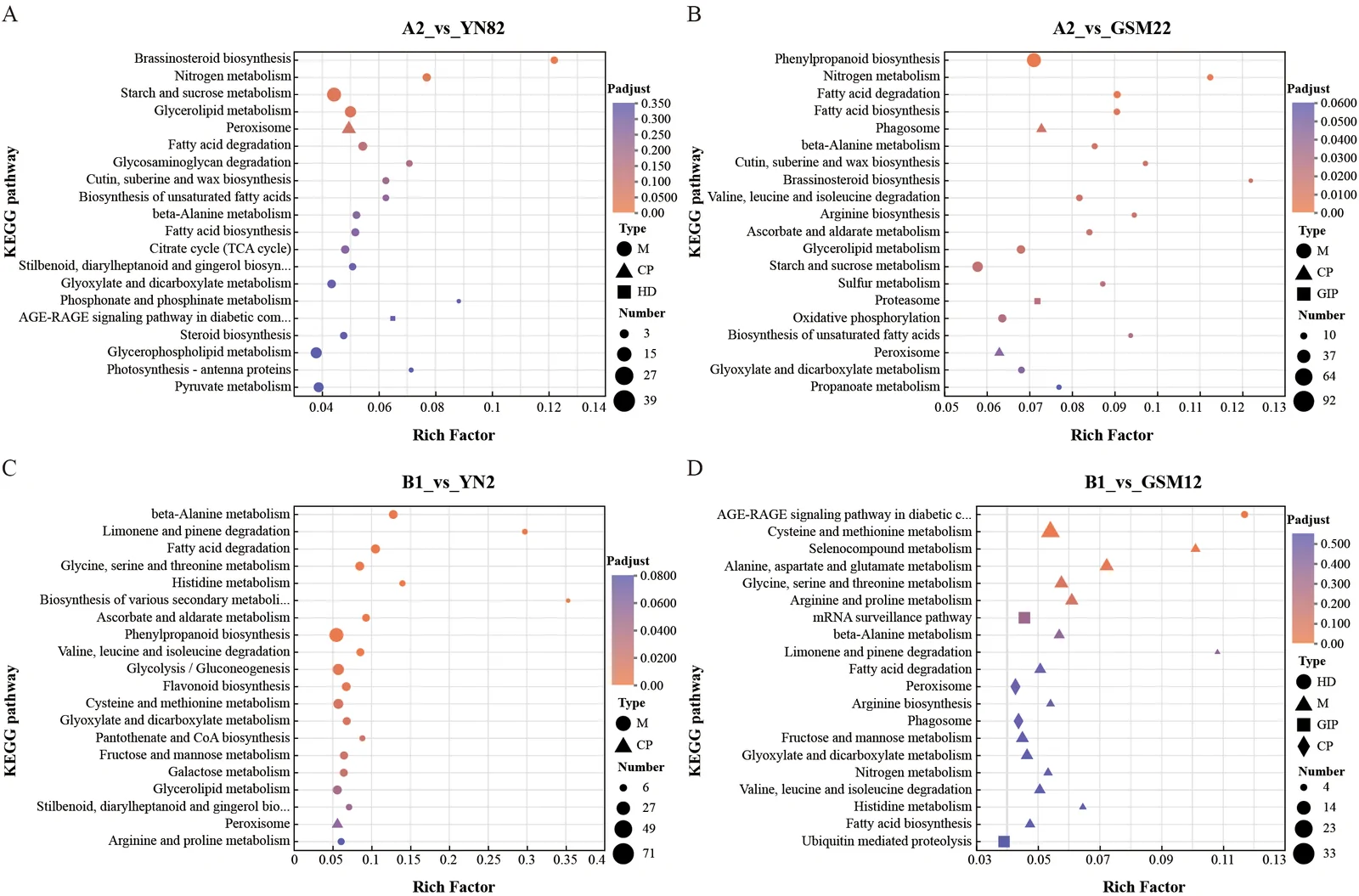
KEGG pathway enrichment analysis of down-regulated differentially expressed genes (DEGs) in hybrid F_1_ vs parents. **(A)** A2 vs YN82; **(B)** A2 vs GSM22; **(C)** B1 vs YN2; **(D)** B1 vs GSM12.

KEGG enrichment analysis indicated that the upregulated genes in the four comparison groups were significantly enriched in pathways such as ribosome (ko03010) and protein processing in the endoplasmic reticulum (ko04141) (**Supplementary Figure 1**). This may indicate a remodeling of basal metabolism in hybrid offspring. Although these pathways are not directly associated with nitrogen uptake phenotypes, existing studies suggest that processes such as ribosome biosynthesis and endoplasmic reticulum protein processing are closely linked to nitrogen metabolism. Furthermore, annotation analysis of the downregulated genes (**Figure 4**) revealed that the downregulated genes in the A2 vs. YN82 and A2 vs. GSM22 comparison groups were significantly enriched in nitrogen metabolism (ko00910) and starch and sucrose metabolism (ko00500) (**Figures 4A, B**). In contrast, the downregulated genes in the B1 vs. YN2 and B1 vs. GSM12 comparison groups were significantly enriched in the phenylpropanoid biosynthesis pathway (ko00940) (**Figures 4C, D**).

**Figure 5:**
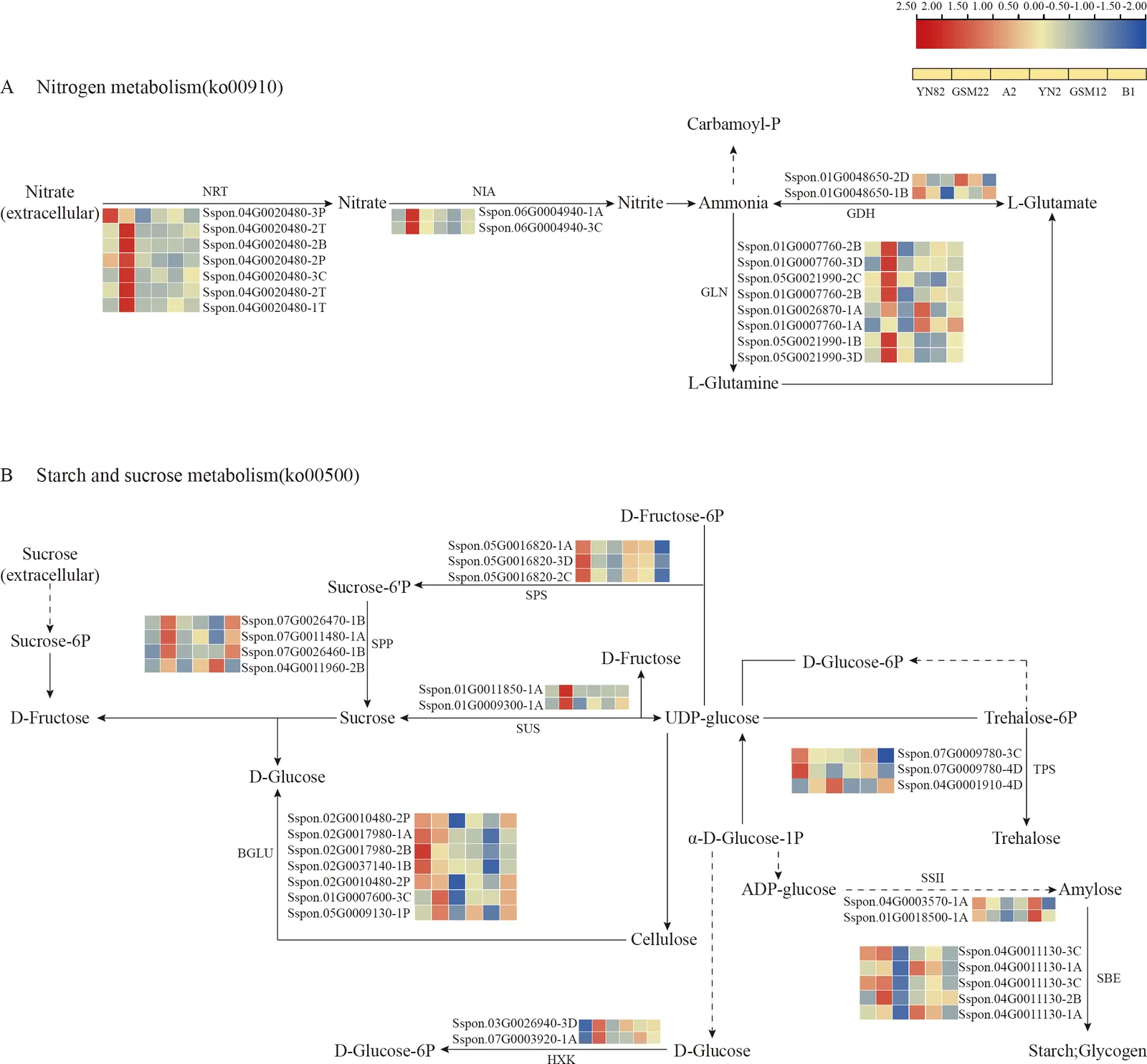
Illustrates the nitrogen metabolism pathway **(A)** and the starch and sucrose metabolism pathway **(B)**, along with the expression patterns of differentially expressed genes (DEGs) within these pathways. Blue signifies downregulated expression, red indicates upregulated expression, and the yellow squares represent the sequence of comparison groups used in the visualization.

The expression patterns of DEGs in the nitrogen metabolism pathway (ko00910; **Figure 5A**) showed that, compared to the parent lines, key genes related to nitrate absorption and assimilation (e.g., *NRT* and *NIA*) were generally down-regulated in the F_1_ generation. This result aligns well with the phenotypic observation that the F_1_ generation exhibits a stronger capacity for ammonium nitrogen uptake compared to nitrate nitrogen. However, counterintuitively, key genes involved in ammonium nitrogen assimilation, such as glutamine synthetase (*GLN*) and glutamate dehydrogenase (*GDH*), also showed a general down-regulation trend in the F_1_ generation. This expression pattern cannot directly explain the enhanced ammonium nitrogen uptake phenotype observed in the progeny. This coordinated down-regulation suggests a strategic metabolic reprogramming in hybrids. Resources appear to be shifted away from the energetically costly nitrate assimilation pathway, potentially favoring the more energy-efficient ammonium utilization route.

As carbon skeletons and energy sources for nitrogen assimilation, the starch and sucrose metabolism pathway (ko00500) also underwent significant reprogramming in the F_1_ generation (**Figure 5B**). Key genes responsible for sucrose synthesis, such as sucrose-phosphate synthase (*SPS5*) and sucrose synthase (*SUS1*), exhibited strong expression in the parent lines but were generally downregulated in the F_1_ generation, suggesting a potential reduction in sucrose synthesis and accumulation. In contrast, several β-glucosidase (*BGLU*) genes and hexokinase genes (*HXK5*), which are involved in sugar hydrolysis, maintained or increased expression levels in certain materials within the F_1_ generation. This overall trend of “weakened synthesis and enhanced hydrolysis and metabolism” likely redirects more carbon flux toward glycolysis, thereby efficiently providing the required carbon skeletons and energy (ATP) for the previously mentioned enhanced ammonium nitrogen assimilation, reflecting a tight coordination between carbon and nitrogen metabolism.

**Figure 6:**
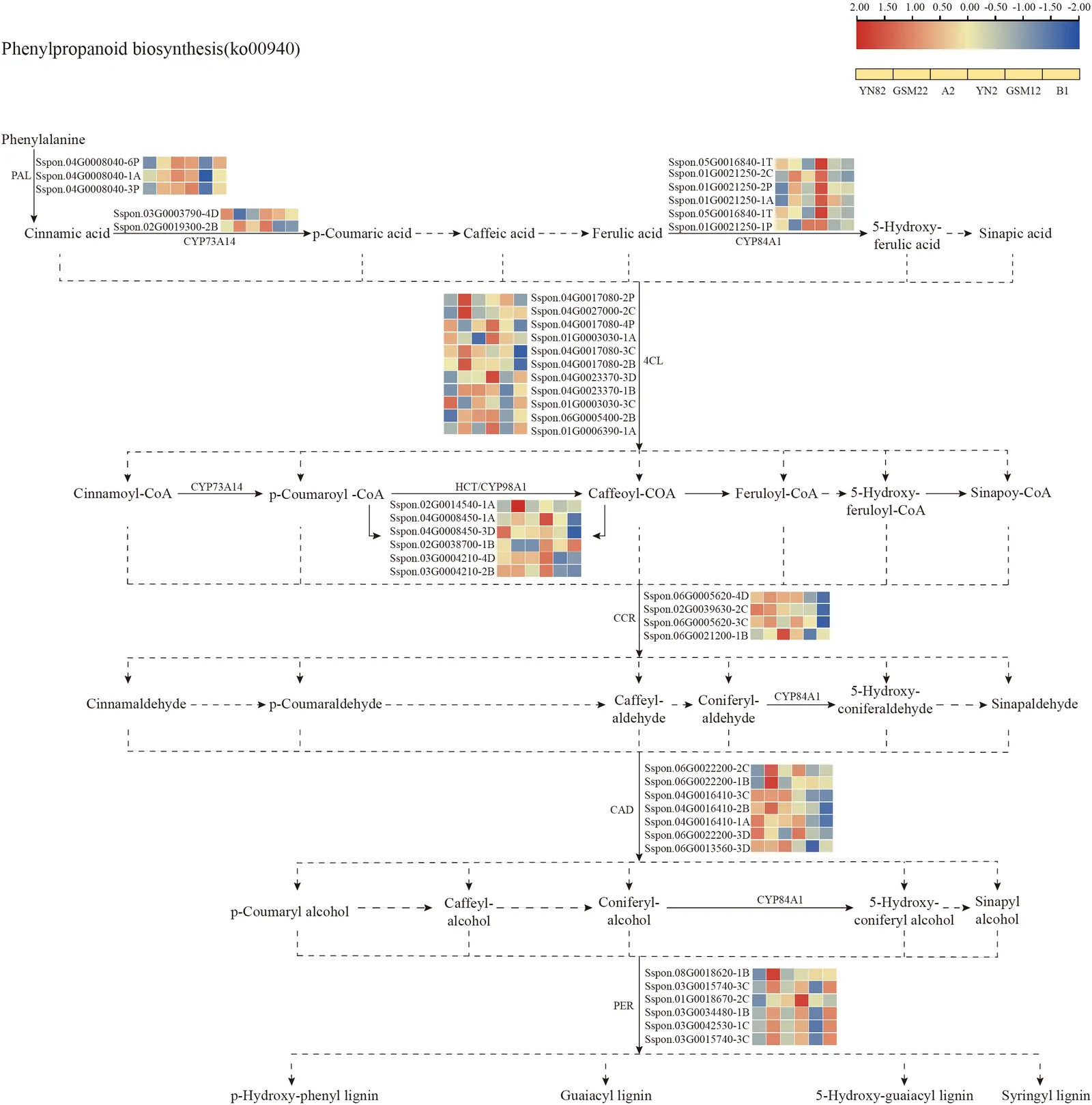
Illustrates the phenylpropanoid biosynthesis metabolic pathway and the expression patterns of differentially expressed genes (DEGs) within it. Blue indicates downregulated expression, red indicates upregulated expression, and yellow squares represent the sequence of comparison groups used in the visualization.

The gene expression patterns within the phenylpropanoid biosynthesis pathway (ko00940; **Figure 6**) reveal the regulation of secondary metabolism. Key enzyme genes at the start of the pathway, such as phenylalanine ammonia-lyase gene *PAL* and ferulate 5-hydroxylase *CYP84A1*, are upregulated in certain F_1_ hybrids, providing sufficient precursors for downstream metabolism. However, genes closely associated with lignin synthesis, such as 4-coumarate-CoA ligase gene *4CL3* and cinnamoyl-CoA reductase gene *CCR1*, exhibit variable expression trends in the F_1_ generation. This pattern suggests that while maintaining the necessary lignin deposition to support root structure, the hybrid progeny may allocate more phenylpropanoid flux to non-lignin branches, such as flavonoid biosynthesis. Additionally, the upregulation of multiple peroxidase genes (*PER*) further indicates the potential role of this pathway in enhancing secondary metabolism, balancing root development, and improving stress defense.

### 3.5 WGCNA analysis based on ^15^N atomic percentage excess

**Figure 7:**
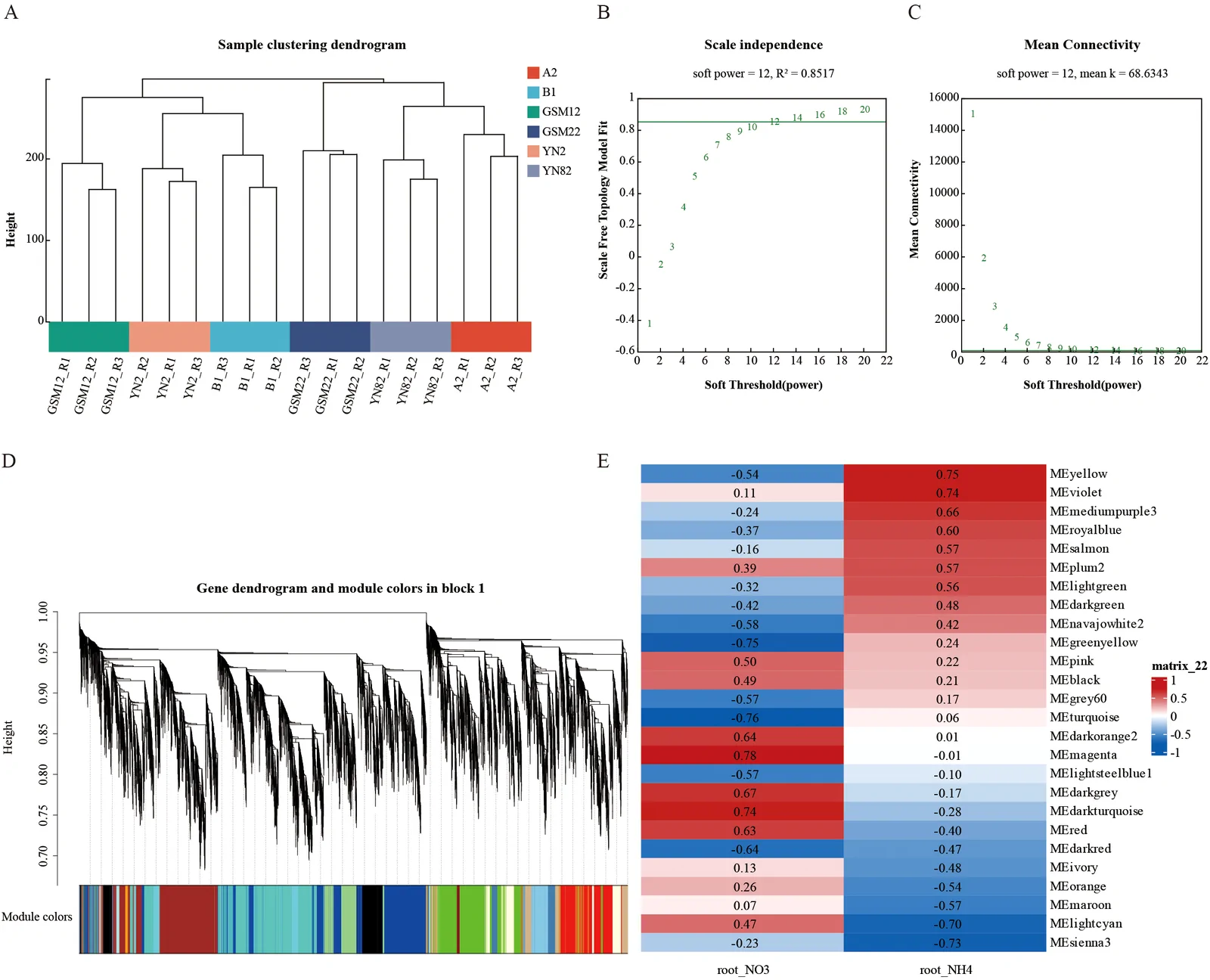
WGCNA analysis: **(A)** Sample clustering; **(B)** Scale-free topology model fit curve; **(C)** Mean connectivity curve; **(D)** Gene clustering and module construction; **(E)** Heatmap of module-phenotype correlations. Only modules with significant correlations (P < 0.05) are shown, and the values inside the squares represent the correlation coefficient r.

To systematically analyze the transcriptional regulatory network underlying root nitrogen absorption, we incorporated transcriptome data from all materials along with phenotypes of nitrate and ammonium nitrogen absorption into a WGCNA (**Figure 7**). Sample clustering analysis revealed that all six samples grouped within their respective clusters without outliers, enabling further analysis (**Figure 7A**). Based on a soft threshold of β = 12 (**Figures 7B, C**), we identified eight gene co-expression modules significantly associated with root nitrogen absorption (**Figures 7D, E**). Among these, four modules (MEyellow, MEviolet, MEsienna3, MElightcyan) showed a highly significant correlation with the markedly enhanced ammonium nitrogen absorption in hybrids (|r| > 0.7, P < 0.05) and were recognized as key modules for subsequent analyzes. Additionally, four modules were found to be significantly associated with nitrate nitrogen, reflecting the relative independence of the regulatory networks for the two nitrogen sources. This also indirectly confirms the high specificity of the ammonium nitrogen-related core modules identified in this study.

### 3.6 Functional annotation of core candidate genes

**Figure 8:**
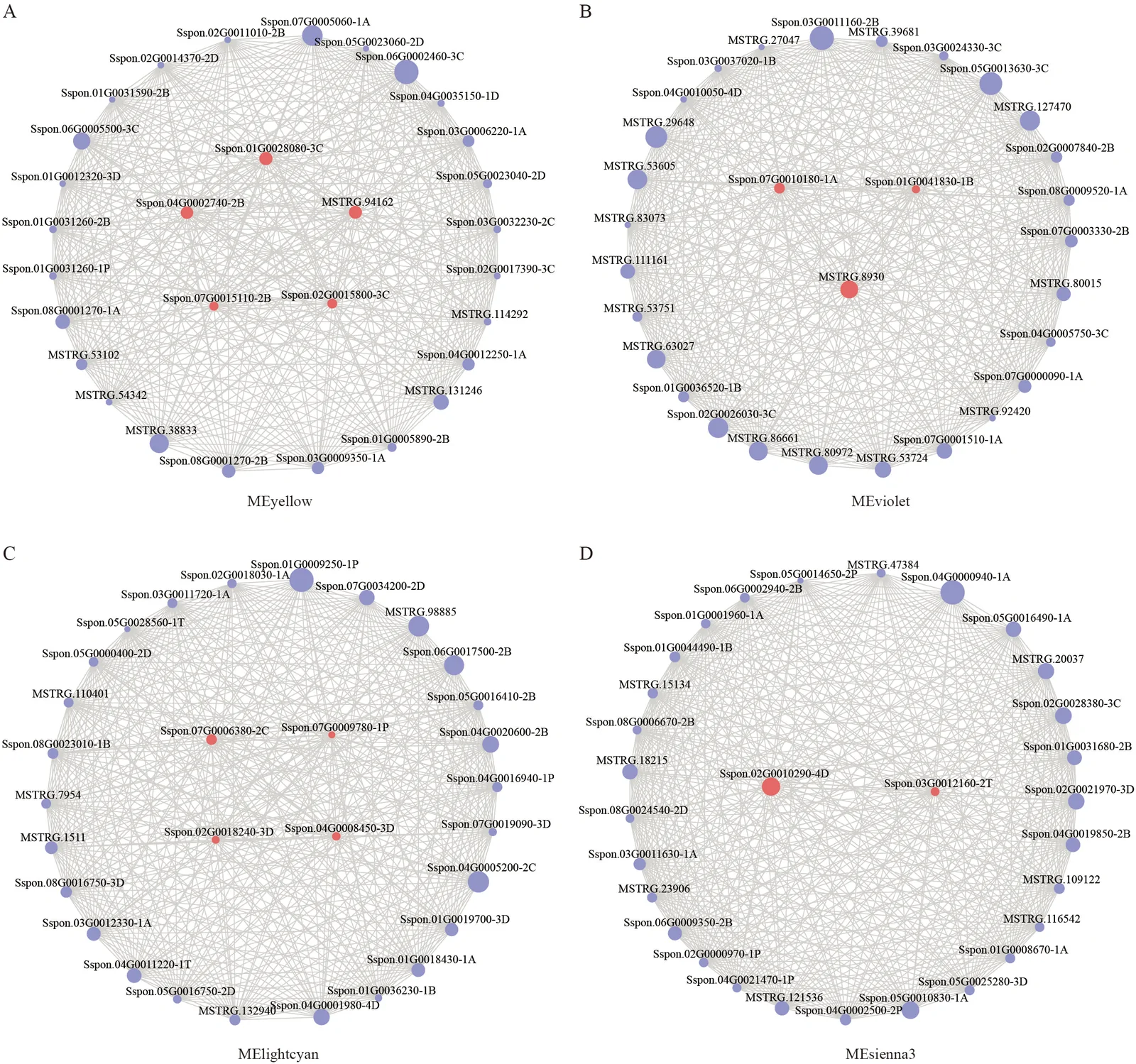
Depicts the interaction network of hub genes within key modules: **(A)** MEyellow module, **(B)** MEviolet module, **(C)** MElightcyan module, and **(D)** MEsienna3 module. Red indicates the core candidate genes, while purple represents the top 30 genes ranked by kME within each module.

To further explore the genes associated with ammonium nitrogen uptake in roots, we conducted an interaction network analysis of the top 30 genes with the highest connectivity in four modules that were significantly correlated with ^15^N atom percentage in root ammonium nitrogen absorption (**Figure 8**). Based on gene functional annotation analysis, 14 genes were identified as core candidate genes (**Table 3**). The results indicate that the proteins encoded by these genes are highly diverse, forming a complex regulatory system that is multi-layered and multi-pathway coordinated. This system primarily consists of the following components: Several key transcription factor families (AP2/EREBP, bHLH, and MYB), which serve as top-tier regulatory hubs within the network; Enzymes directly involved in nitrogen assimilation and amino acid metabolism (glutamate decarboxylase *GAD* and glutamyl-tRNA synthetase), which are responsible for nitrogen fixation and conversion; Key enzymes in carbon metabolism pathways (trehalose-6-phosphate phosphatase *TPP*, trehalose-6-phosphate synthase *TPS* and glycogen phosphorylase *GlgC*), which provide carbon skeletons and energy for nitrogen metabolism; Critical enzymes in the phenylpropanoid biosynthesis pathway (hydroxycinnamoyl transferase *HCT*, ferulate 5-hydroxylase *CYP84A1*, and aldehyde dehydrogenase *REF1*), which are closely associated with root development and structural reinforcement. This study highlights the intricate and cooperative regulatory network underlying ammonium nitrogen uptake and related metabolic processes in roots.

**Table:** Table 3 Functional annotation of core candidate genes.

Module	Gene ID	Annotation	KEGG pathway	PFAMs
Yellow	MSTRG.94162	Ethylene-responsive transcription factor 4	--	AP2
Yellow	Sspon.04G0002740-2B	Ethylene-responsive transcription factor 4	–	AP2
Yellow	Sspon.01G0028080-3C	Transcription factor BHLH148	–	HLH
Yellow	Sspon.07G0015110-2B	Glutamate decarboxylase 1	Alanine, aspartate and glutamate metabolism	Pyridoxal_deC; Aminotran_5
Yellow	Sspon.02G0015800-3C	Probable trehalose-phosphate phosphatase 7	Starch and sucrose metabolism	Trehalose_PPase
Violet	Sspon.07G0010180-1A	Dehydration-responsive element-binding protein	–	AP2
Violet	MSTRG.8930	Glucose-1-phosphate adenylyltransferase large subunit	Starch and sucrose metabolism	–
Violet	Sspon.01G0041830-1B	Glutamate--tRNA ligase, cytoplasmic	Aminoacyl-tRNA biosynthesis	tRNA-synt_1c
Lightcyan	Sspon.02G0018240-3D	Transcription factor MYB73	–	Myb_DNA-binding
Lightcyan	Sspon.07G0009780-1P	Alpha, alpha-trehalose-phosphate synthase	Starch and sucrose metabolism	Trehalose_PPase; Hydrolase_3
Lightcyan	Sspon.04G0008450-3D	Hydroxycinnamoyltransferase 2	Phenylpropanoid biosynthesis	Transferase
Lightcyan	Sspon.07G0006380-2C	Flavonoid 3’-monooxygenase CYP75B4	Flavonoid biosynthesis	p450
Sienna3	Sspon.02G0010290-4D	Transcription factor 25	–	Tcf25
Sienna3	Sspon.03G0012160-2T	Aldehyde dehydrogenase family 2 member C4	Aldehyde dehydrogenase family 2 member C4	Aldedh

### 3.7 RT-qPCR validation

**Figure 9:**
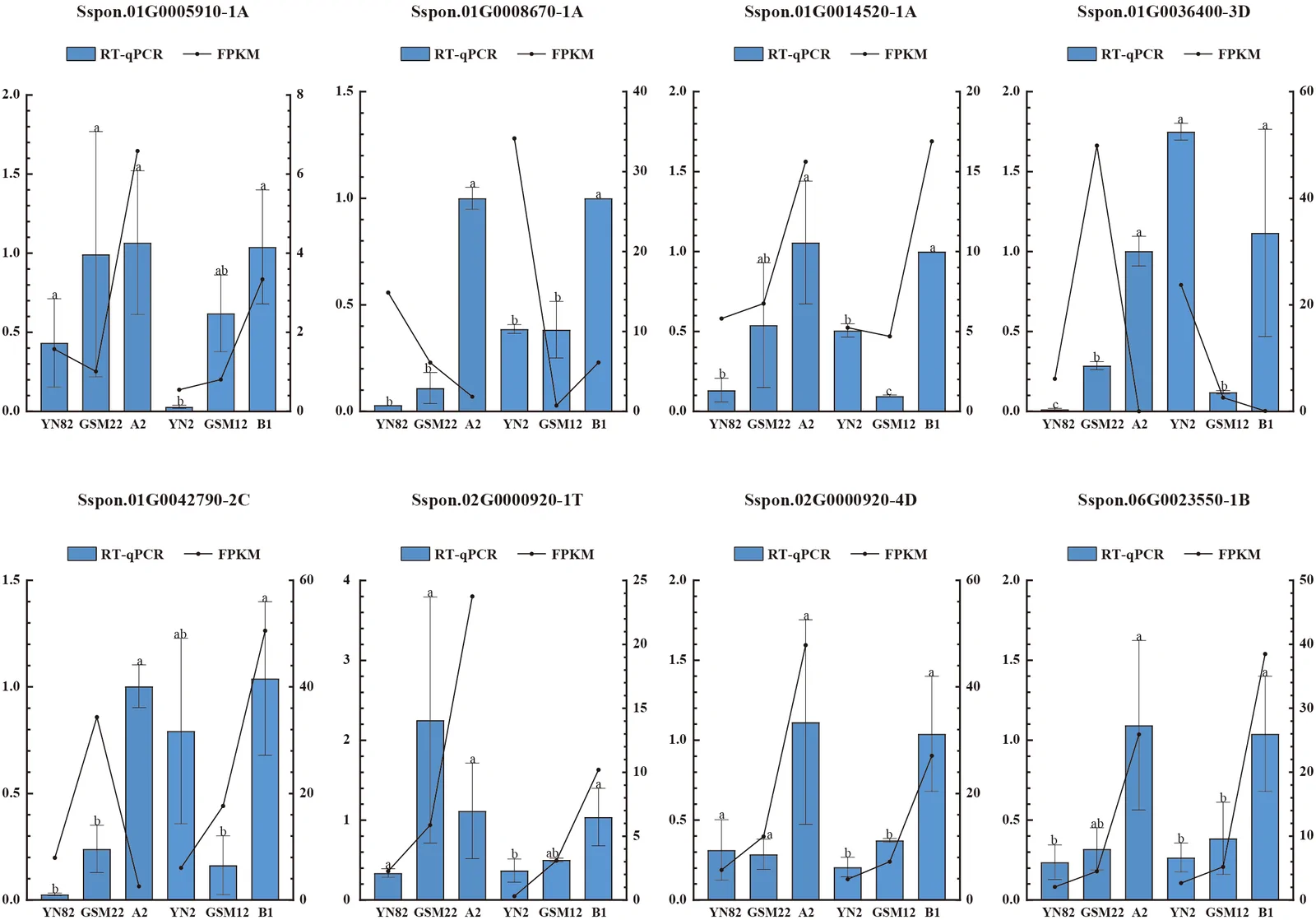
Presents the expression results of eight selected genes determined using RT-qPCR and RNA-Seq analysis. The RT-qPCR data are shown as mean ± standard error from three independent experiments. Bars labeled with different letters indicate significant differences at the p < 0.05 level. The left vertical axis of the bar chart represents RT-qPCR values, while the right vertical axis indicates FPKM values.

To validate the reliability of the transcriptome results, eight genes were randomly selected for RT-qPCR analysis (**Figure 9**). These genes include *Sspon.01G0005910-1A*, *Sspon.01G0008670-1A*, *Sspon.01G0014520-1A*, *Sspon.01G0036400-3D*, *Sspon.01G0042790-2C*, *Sspon.02G0000920-1T*, *Sspon.02G0000920-4D*, and *Sspon.06G0023550-1B*. The results demonstrated that the relative expression trends measured by RT-qPCR were largely consistent with the transcriptome sequencing data (FPKM values) for most genes, thereby confirming the overall accuracy of our transcriptome dataset. Notably, any instances of quantitative discrepancy (e.g., for gene Sspon.01G0036400-3D) highlight the importance of such orthogonal validation and may reflect the technical differences between the two methods.

## 4 Discussion

### 4.1 Specific enhancement of ammonium uptake: an energetically favorable phenotype

This study presents clear physiological evidence that intraspecific hybridization in *S.* sp*ontaneum* specifically and significantly enhances root ammonium (¹^5^NH_4_^+^) uptake capacity, without a corresponding increase in nitrate (¹^5^NO_3_⁻) uptake (**Figure 1**). This distinct phenotypic trait aligns with the classification of sugarcane as an “ammonium-preferring” crop and can be primarily attributed to energy economy (Chen et al., 2024; Zhan et al., 2025). Ammonium assimilation requires substantially less ATP than nitrate reduction and assimilation—a well-documented metabolic constraint in plants (Liu et al., 2017; Hou et al., 2021; The et al., 2021). Therefore, the hybrid’s enhanced preference for ammonium reflects a more efficient nitrogen acquisition strategy, likely improving resource allocation when metabolic energy is limited (Nacry et al., 2013; Chen et al., 2020; Amoah et al., 2025). This physiological adaptation is further supported at the molecular level: WGCNA revealed that gene modules most strongly associated with high ammonium uptake were enriched in pathways related to energy production and protein synthesis, such as oxidative phosphorylation and ribosome biogenesis. Existing studies have indicated that ribosomal-associated pathways, although not directly linked to nitrogen uptake phenotypes, maintain a close relationship with these traits. As the site of protein synthesis, ribosomes have functions that are directly connected to cellular nitrogen metabolism, with nitrogen serving as a fundamental constituent of macromolecules such as proteins and nucleic acids (Fan et al., 2024). For instance, in cyanobacteria subjected to prolonged phosphate starvation, nitrogen metabolism and ribosome biogenesis are among the most significantly altered pathways; phosphate starvation leads to an increase in ribosomal quantity but a decrease in their activity, thereby impairing protein synthesis and indirectly affecting nitrogen assimilation and utilization (Wei et al., 2023). In studies on nitrite-oxidizing bacteria (NOB), a side-stream free ammonia treatment system was established to simulate NOB inhibition. It was found that dysregulated ribosome biogenesis contributes to NOB suppression, which in turn promotes nitrite accumulation—highlighting ribosomal regulation as a key factor in the nitrogen cycle (Xiong et al., 2023). In wheat, nitrogen treatment alters gene expression and protein levels associated with protein synthesis and protein body development, indicating that ribosomes and the endoplasmic reticulum play central roles in the efficient utilization of nitrogen for protein synthesis (Yu et al., 2017). These findings indicate that the hybrid state is systemically optimized to support the energetic and biosynthetic demands of preferential ammonium utilization (Chen et al., 2020; Fu et al., 2023).

### 4.2 Systemic transcriptional reprogramming underpins metabolic co-optimization

The observed phenotypic advantage is not attributable to a single gene but emerges from a genome-wide transcriptional reprogramming that orchestrates a synergistic reconfiguration of interconnected primary and secondary metabolic networks (Liu et al., 2017; Chen et al., 2020; Pélissier et al., 2024; Khan et al., 2025). This finding reinforces the prevailing concept that complex agronomic traits like nitrogen use efficiency (NUE) are systems-level properties, arising from the integrated operation of multiple pathways rather than isolated genetic effects. Our transcriptomic analysis captures this integration. In the hybrids, a coordinated downregulation was observed across the nitrogen metabolism pathway (ko00910), encompassing genes for nitrate transport (*NRT*), nitrate reduction (*NIA*), and core ammonium assimilation enzymes like glutamine synthetase (*GLN*) (**Figure 5A**). We interpret this broad pattern not as a suppression but as a signature of strategic metabolic streamlining, potentially reducing flux through less favored or redundant pathways to increase overall efficiency (Yan et al., 2023; Wu et al., 2024).

Concurrently, a profound and complementary rewiring of carbon metabolism was evident. Within the starch and sucrose metabolism pathway (ko00500), genes associated with sucrose synthesis and storage (e.g., *SPS5*, *SUS1*) were downregulated, whereas those facilitating sugar hydrolysis and glycolytic entry (e.g., *BGLU*, *HXK*) were upregulated (**Figure 5B**). This “shift from storage to mobilization” likely channels carbon flux toward glycolysis and the TCA cycle, ensuring a ready supply of essential carbon skeletons (e.g., 2-oxoglutarate) and ATP for the GS/GOGAT cycle responsible for ammonium assimilation. This precise demand-driven adjustment of carbon catabolism exemplifies the critical, tight coupling—or “matrix effect”—between carbon and nitrogen metabolism that governs plant growth and resource partitioning (Pélissier et al., 2024). Furthermore, the observed reconfiguration of the phenylpropanoid biosynthesis pathway (ko00940), marked by the upregulation of early genes like *PAL* and *CYP84A1*, indicates an adjustment that may simultaneously support root structural integrity through lignin deposition and modulate secondary metabolism, thereby indirectly creating a root environment more conducive to nutrient acquisition.

### 4.3 A multi-tiered gene co-expression network drives the optimized phenotype

**Figure 10:**
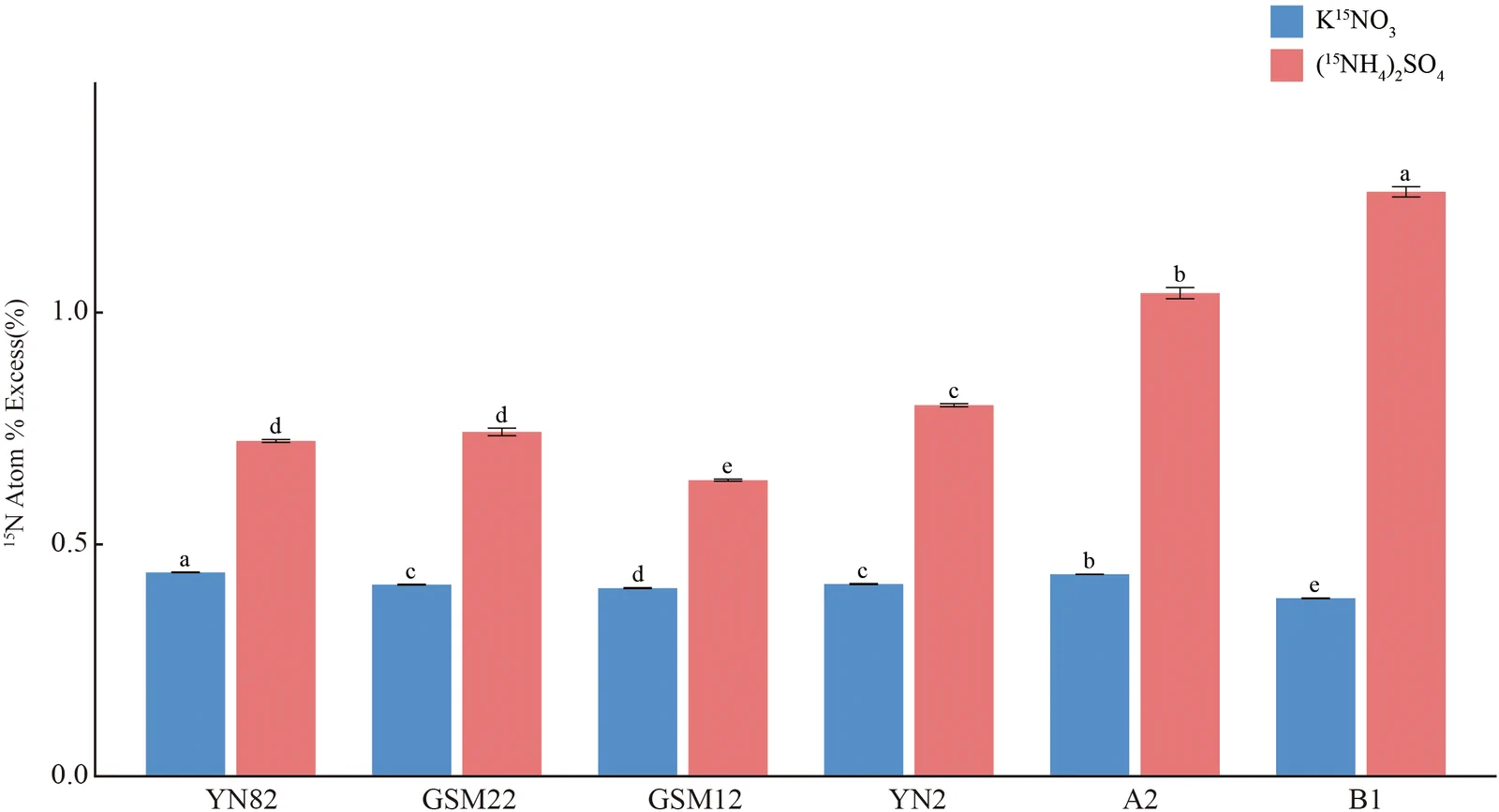
Nitrogen uptake kinetics in leaves of the six sugarcane materials. Data are presented as mean ± SD (n = 3). Statistical significance was determined by one-way ANOVA, and different letters above the bars indicate significant differences at *P* < 0.05.

To transition from describing system-level correlations to identifying potential regulatory architectures, we employed WGCNA. This approach successfully distilled the complex transcriptional landscape into a tractable gene co-expression network exhibiting a highly significant correlation with the ammonium uptake phenotype, demonstrating its power to elucidate the genetic foundations of complex traits. From this network, we pinpointed 14 core candidate genes that constitute a coherent, multi-tiered regulatory module (**Table 3**, **Figure 8**). This module is functionally stratified, encompassing several interconnected layers. At the transcriptional regulatory level, hub genes encoding AP2/EREBP, bHLH, and MYB family transcription factors are positioned to act as master regulators, potentially sensing nitrogen or energy status to initiate the broader transcriptional cascade. At the core of metabolic integration, key enzymes such as Glutamate Decarboxylase (*GAD*) underscore the centrality of nitrogen metabolism within the network. The inclusion of trehalose-6-phosphate phosphatase and synthase (*TPP*, *TPS*) is particularly noteworthy, as trehalose-6-phosphate (*T6P*) is a well-established global signaling molecule that integrates sugar availability with growth and stress responses (Zhang et al., 2009; Lin et al., 2019; Meitzel et al., 2019; Fichtner and Lunn, 2021; Morales-Herrera et al., 2023; Fichtner, 2025), making it a prime candidate for coordinating the observed carbon-nitrogen synergy. Finally, connecting internal metabolic states to developmental outputs, genes such as hydroxycinnamoyltransferase (*HCT*) and *CYP84A1* link the network to phenylpropanoid metabolism, thereby influencing root development and structural fortification. This correlation is of significant importance, as the current study demonstrates that the differential expression of phenylpropanoid biosynthesis genes in the progeny exerts a substantial influence on nitrogen uptake efficiency. Physiological data indicate that not only has the ammonium nitrogen absorption capacity of the root system improved, but the ammonium nitrogen content accumulated in the leaves has also increased significantly, whereas the uptake status of nitrate nitrogen remains unclear (**Figure 10**). Furthermore, the differential expression of these genes enhances the transport efficiency of ammonium nitrogen from the root system to the leaves in the *S.* sp*ontaneum* F_1_ generation, thereby improving the progeny’s capacity for ammonium nitrogen assimilation. Despite the down-regulation of phenylpropanoid biosynthesis-related genes in the offspring, there is still a significant increase in ammonium nitrogen absorption, which presents a compelling mechanistic hypothesis: the heterosis in *S.* sp*ontaneum* hybrids may result from superior signal integration and resource coordination at the transcriptional, metabolic, and developmental levels in the offspring (Park et al., 2024; Sowmiya et al., 2024).

### 4.4 Considerations of environmental interactions and trait trade-offs

It should be noted that the present study was conducted under controlled conditions at the seedling stage, using pot cultures with regulated nitrogen sources. This experimental setup provides a necessary foundation for precisely dissecting the intrinsic mechanisms of intraspecific hybridization. However, it also implies that the observed heterosis in ammonium uptake may be modulated by complex field conditions, such as the soil ammonium/nitrate ratio, temperature fluctuations, and rhizosphere microbial activity, all of which could influence nitrogen acquisition in hybrid progenies. Therefore, validating the selected core germplasms (A2, B1) across multiple field environments with diverse nitrogen management practices constitutes an indispensable next step toward translating these findings into practical breeding outcomes.

Furthermore, the specifically enhanced ammonium uptake observed in hybrid progeny, coupled with the coordinated downregulation of the nitrate assimilation pathway, raises questions regarding potential physiological trade-offs. We propose that the transcriptional reprogramming observed does not reflect a loss of function but rather a strategic optimization of resource allocation. The more energy-intensive nitrate reduction process is streamlined, and the conserved energy is redirected toward restructuring carbon metabolism, thereby facilitating more efficient ammonium assimilation. Similarly, variations in gene expression within the phenylpropanoid biosynthesis pathway may signify an optimized allocation between lignin synthesis and the production of other secondary metabolites, balancing the dual demands of root structural integrity and environmental adaptation. In summary, hybridization appears to trigger a systemic optimization centered on improving the overall efficiency of the carbon–nitrogen metabolic network, rather than enhancing isolated traits. Future studies that compare hybrids and their parental lines across a broader spectrum of physiological parameters will provide a more comprehensive evaluation of this heterosis phenomenon.

### 4.5 Implications for a paradigm shift in breeding nitrogen-efficient crops

Our findings carry significant implications for the future of crop improvement, advocating for a strategic paradigm shift in breeding for enhanced NUE. Moving beyond the introgression or overexpression of single major-effect genes (e.g., high-affinity *AMT/NRT* transporters)—an approach often plagued by context dependency and inconsistent results—we highlight the substantial potential of leveraging intraspecific hybridization within untapped wild germplasm to systemically aggregate favorable alleles across synergistic gene networks. The core regulatory network identified in this study effectively constitutes a “functional module” for ammonium use efficiency. This reconceptualization of the breeding target, from a single gene to a synergistic gene set, enables two advanced and complementary strategies for molecular breeding. First, the core candidate genes provide a targeted suite of molecular markers for implementing multi-gene, module-based Marker-Assisted Selection (MAS). This allows for the selection of progeny that have inherited the beneficial haplotype across the entire functional module, thereby increasing the probability of capturing the robust, systems-level phenotype. Second, key network nodes, especially signaling components like TPP/TPS and integrative metabolic enzymes like GAD, offer high-value, precision targets for genetic engineering aimed at fine-tuning the carbon-nitrogen interface to improve NUE with greater predictability (Morabito et al., 2021; Noda et al., 2024). In conclusion, our integrated analysis reveals that heterosis for nitrogen efficiency in *S.* sp*ontaneum* is a systems-level phenomenon. It is driven by a specific shift towards energy-efficient ammonium uptake, supported by a reprogrammed carbon metabolism that ensures substrate supply, and regulated by a multi-layered gene co-expression network. This work delivers not only a valuable set of candidate gene resources but also a refined conceptual framework—one that embraces biological complexity and synergy—for guiding the development of next-generation, nitrogen-efficient sugarcane varieties.

## 5 Conclusion

The hybrids of *Saccharum* sp*ontaneum* achieved a significant enhancement in nitrogen accumulation through the specific improvement of root ammonium nitrogen absorption efficiency. The molecular foundation of this nitrogen-efficient phenotype lies in the systematic synergistic optimization of multiple pathways, including nitrogen metabolism (ko00910), starch and sucrose metabolism (ko00500), and phenylpropanoid biosynthesis (ko00940). Additionally, the transcriptional reprogramming triggered by hybridization activated a multilayered regulatory network comprising 14 core candidate genes. This network integrates key biological processes, ranging from transcriptional regulation and nitrogen assimilation to carbon skeleton supply and root architecture construction. These findings reveal a novel perspective of “systematic optimization” in elucidating the mechanisms underlying the nitrogen efficiency traits of wild germplasm resources. They not only offer new insights into the complex regulatory networks involved in plant nitrogen efficiency but also provide a robust theoretical foundation and valuable genetic resources for germplasm innovation and molecular marker development in nitrogen-efficient sugarcane breeding.

## Data Availability

The datasets presented in this study can be found in online repositories. The raw reads have been deposited in the Genome Sequence Archive (GSA, https://ngdc.cncb.ac.cn/gsa/) under accession number CRA034490. All other generated datasets are provided within the article and **Supplementary Information Files**.

## Supplementary material

Supplementary Figure 1 KEGG pathway enrichment analysis of up-regulated differentially expressed genes (DEGs) in hybrid F1 vs parents. **(A)** A2 vs YN82; **(B)** A2 vs GSM22; **(C)** B1 vs YN2; **(D)** B1 vs GSM12.
