# Supplementary figures and images for: Transcriptional reprogramming and co-expression network underlying enhanced ammonium uptake in intraspecific hybrids of Saccharum spontaneum
Source: Front Plant Sci. 2026 Feb 25;17:1758719. doi: 10.3389/fpls.2026.1758719 (PMC12975887; doi:10.3389/fpls.2026.1758719)

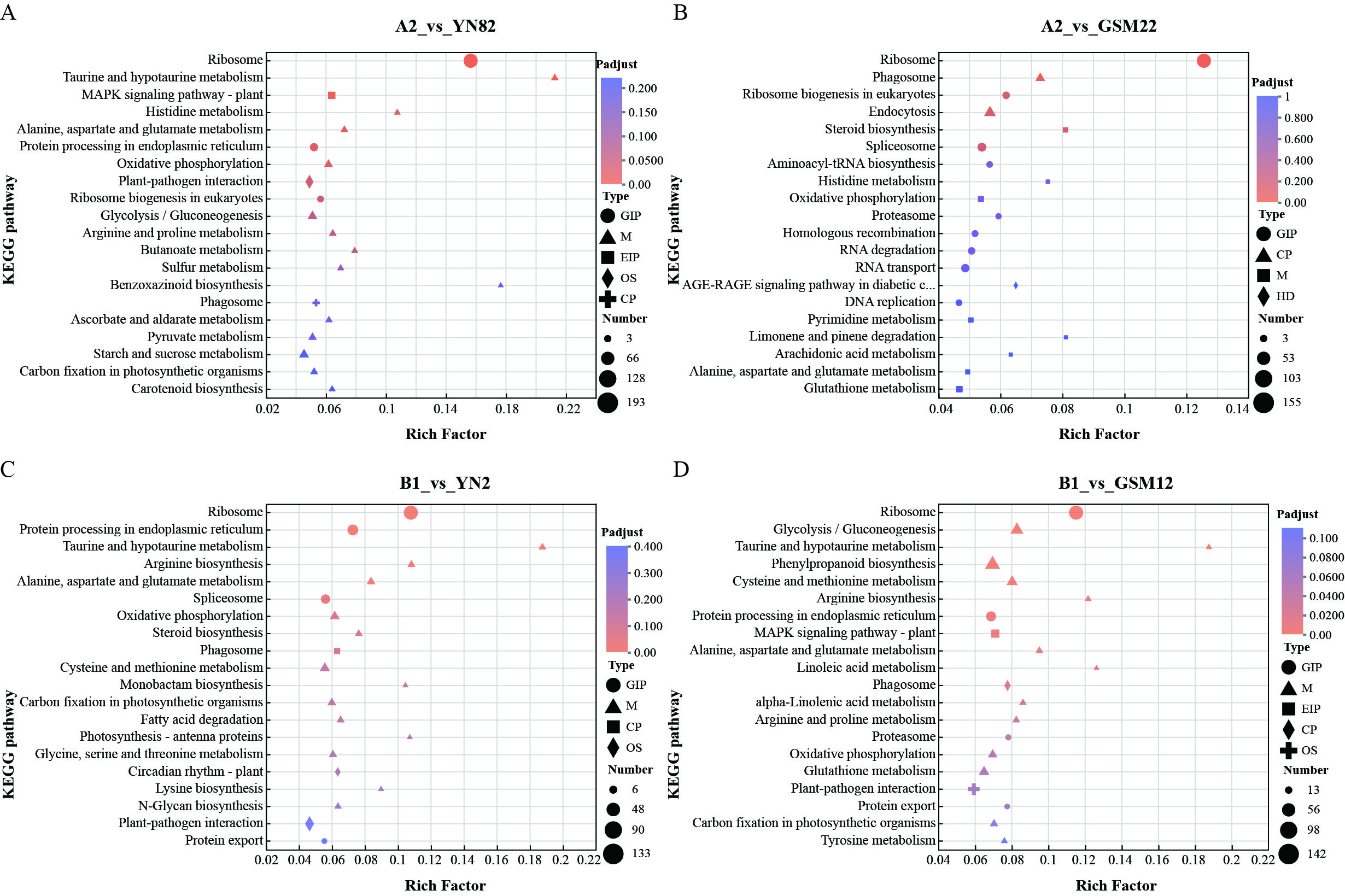

Supplement: Supplementary Figure 1 — KEGG pathway enrichment analysis of up-regulated differentially expressed genes (DEGs) in hybrid F1 vs parents. (A) A2 vs YN82; (B) A2 vs GSM22; (C) B1 vs YN2; (D) B1 vs GSM12. [file Image1.jpeg]
